# Verbal Creativity Is Correlated With the Dynamic Reconfiguration of Brain Networks in the Resting State

**DOI:** 10.3389/fpsyg.2019.00894

**Published:** 2019-04-24

**Authors:** Qiuyang Feng, Li He, Wenjing Yang, Yao Zhang, Xinran Wu, Jiang Qiu

**Affiliations:** ^1^Faculty of Psychology, Southwest University, Chongqing, China; ^2^Key Laboratory of Cognition and Personality (SWU), Ministry of Education, Chongqing, China

**Keywords:** creativity, resting state, dynamic reorganization, brain networks, neural mechanism

## Abstract

Creativity is the foundation of human culture. All inventions and innovations in history rely upon us to break with the traditional thinking and create something novel. A number of neuroimaging studies have explored the neural mechanism of creativity. However, a majority of researches have focused only on the stationary functional connectivity in resting-state fMRI and task-related fMRI, neglecting the dynamic variation of brain networks. Here, we used dynamic network analysis to investigate the relation between the dynamic reorganization of brain networks and verbal creativity in 370 healthy subjects. We found that the integration of the left lingual gyrus and left middle temporal gyrus (MTG) in default mode network (DMN) and the integration of the DMN and cerebellum, frontoparietal task control network (FPTC) and auditory network (Aud) showed positive correlation with verbal creativity performance. In addition, the recruitment of the bilateral postcentral gyrus from the sensory/somatomotor network (SMN) and the recruitment of the SMN in general displayed a significant correlation with verbal creativity scores. Taken together, these results suggested that the dynamic reorganization among the brain networks involved multiple cognitive processes, such as memory retrieval, imaginative process, cognitive control – these are all important for verbal creativity. These findings provided direct evidence that verbal creativity was related to the dynamic variation of brain mechanism during resting-state, extending past research on the neural mechanism of creativity. Meanwhile, these results bought about new perspectives for verbal creative training and rehabilitation training of depression.

## Introduction

Creativity is the foundation of human culture. All inventions and innovations in history rely upon us to break with the traditional thinking and create something novel. Creativity is generally defined as the capability of generating original and useful products (Runco and Jaeger, 2012). Based on the definition, the psychometric measurements about creative performance mostly depend on divergent thinking (DT) tasks, in which individuals are asked to think of several possible solutions to open questions (Runco and Acar, 2012). The most common DT task is the Alternative Uses Task (AUT), in which subjects should generate various suitable and creative usages of familiar items, such as a tin (e.g., “eating”) (Guilford, 1967). Then the answers are scored for three dimensions including fluency (the amount of possible solutions), flexibility (the amount of solutions classifications), and originality (the unusual of solutions) (Guilford, 1967).

Recently, neural mechanism has increasingly been the hot topic of research in creativity. Although there is a lack of agreement about the neural mechanism of creativity, there was some overlap in brain activated regions, involving the prefrontal cortex (PFC) (Arden et al., 2010), middle temporal gyrus (MTG) (Mark et al., 2004; plosbiology, 2004), posterior cingulate cortex (PCC) (Dietrich and Kanso, 2010; Zhang H. et al., 2014), and posterior parietal cortex (PPC) (Howard-Jones et al., 2005; Mashal et al., 2007). A meta-analysis based on the activation likelihood estimation showed that the lateral PFC, PPC, and left MTG were typically activated under DT tasks (Xin et al., 2015). Furthermore, Wei et al. (2014) reported that the great DT tasks performance was related to stronger strength of functional connectivity (FC) between the medial PFC and the MTG during resting-state. Meanwhile, some studies showed the results of brain structure in creativity. For example, better creativity performance was related to increased gray matter thickness in the right PCC (Clark et al., 2013). Lower gray matter thickness in the lingual gyrus was correlated to higher DT tasks scores (Mula et al., 2016).

However, the process of generating novel ideas is not only involved in some specific brain regions. Rather, it mostly depended on cooperation among multiple brain networks (Dietrich, 2007; Dietrich and Kanso, 2010). Recently, research has also focused on the large-scale functional networks in creativity, and revealed two key networks: default mode network (DMN) and frontoparietal network (FPN). The DMN comprised of the medial PFC, the PCC, the precuneus, the MTL, and bilateral inferior parietal lobes (IPL), which were often activated in DT tasks (Fink et al., 2010; Sun et al., 2013). This network was consistently activated in the absence of external task demands and deactivated during an external task (Kim et al., 2014; Luo et al., 2015). As it was in the absence of cognitive control (van den Heuvel and Hulshoff Pol, 2010), the DMN may be a benefit to the creative thoughts to enter our consciousness (van den Heuvel and Hulshoff Pol, 2010; Beaty et al., 2014). The FPN was associated with the top-down control of cognitive and executive. It included lateral prefrontal and anterior inferior parietal regions, which were also continually activated in creativity tasks (Abraham et al., 2012; Zabelina and Andrews-Hanna, 2016). Regions of the FPN played a critical roles in the process of generating novel ideas. For example, great cognitive manipulation facilitated the concentration of thoughts details and the assessment of the usefulness of creative ideas (Carter and Veen, 2007; Ellamil et al., 2012).

In addition, a great deal of recently published papers have revealed the relationship between FC of the DMN and FPN and creative performance (Beaty et al., 2014; Shi et al., 2018b). For example, Zhu et al. (2017) showed that the coupling between the DMN and FPN was obviously associated with creativity scores. However, a majority of research has focused only on the stationary functional connectivity in resting-state fMRI and task-related fMRI. The latest studies have revealed that brain network organizations varied more and less at any time (Zhang X. et al., 2014), which makes it possible to study the fluctuation characteristics of the brain networks over time by following the immediate interaction patterns among brain regions (Madhyastha et al., 2015; Tagliazucchi and Laufs, 2015). To date, most studies about the association between the brain networks and creativity overlooked the dynamic reorganization of the brain networks. Intriguingly, the flexibility among the networks has been proved to be associated with cognitive flexibility (Bassett et al., 2011; Bertolero et al., 2015). Dietrich (2004) showed that creativity was the essence of cognition flexibility, which could promote change of thinking to adapt environmental variation, and bring about the production of creative ideas and invention (Dietrich, 2004; Barbey et al., 2013). All in all, the cognition flexibility in creative people may reflect in dynamic changeability and reorganization among the brain networks during the resting state. Therefore, the present study investigated the correlation between the dynamic reorganization of brain networks and creativity.In the current study, the participants completed an AUT and intelligence test after measured neuroimaging. Given that the past studies had indicated that creative thinking was related to the cooperation between the DMN and FPN (Beaty et al., 2014, 2015; Zhu et al., 2017; Shi et al., 2018b), we hypothesized that the dynamic communication between the DMN and FPN might be also related to creative performance. To address this hypothesis, we used dynamic network construction and multilayer community detection to explore the relationship between dynamic communication patterns among the brain networks and creative performance.

## Materials and Methods

### Participants

A total of 410 healthy undergraduates were recruited from Southwest University in China. Seventeen participants did not complete the behavioral test, and fifteen participants did not participate in the scanning. Eight subjects were excluded because the maximal head motion between volumes in each direction exceeded 3 mm or rotation about each axis exceeded 3°. Then, 370 undergraduates (166 males; mean age = 19.98, *SD* = 1.27) were included in the next analyses. Based on a self-report questionnaire before scanning, none of them had reported a history of neurological or mental illness or substance abuse. All subjects signed the written informed consents. This study was approved by the Institutional Review Board of Southwest University Imaging Center.

### Assessment of Creativity

We measured creative cognition using AUT from the verbal form of the Torrance Tests of Creative Thinking (Torrance, 1974). Subjects were asked to list as many interesting and unusual uses as possible for a cardboard box using paper and pencil within 10 min. We calculated three different creative dimensions scores, including (1) originality (the ability to produce uncommon ideas), (2) flexibility (the ability to generate more different kinds of ideas), and (3) fluency (the ability to generate more meaningful ideas). Three trained raters assessed responses of all participants based on previous guidance (Chen et al., 2014), and the inter-rater correlation coefficient was adequate (ICC > 0.90).

### Assessment of General Intelligence

As a test of general intelligence, all participants completed the Combined Raven’s Test (CRT). The CRT consists of Raven’s colored progressive matrix (A, B, and AB sets) and Raven’s standard progressive matrix (C, D, and E sets), which have been widely used in Chinese populations with a high degree of reliability and validity in measuring general intelligence (Wang et al., 2007; Tang et al., 2012). The index of general intelligence was calculated by the total number of correct answers given in 40 min.

### Data Acquisition and Preprocessing

Functional imaging data were collected on a Siemens 3T Trio scanner (Siemens Medical Systems, Erlangen, Germany). Resting-state fMRI was conducted with the following EPI sequences: TR = 2000 ms, TE = 30 ms, field of view (FOV) = 220 × 220, flip angle = 90°, slices = 32, thickness = 3 mm, slice gap = 1 mm, voxel size = 3.4 × 3.4 × 4 mm^3^. The scanning lasted 8 min and contained 242 volumes. During the scanning, all participants were asked to close eyes and rest, but not to fall asleep.

The fMRI data were preprocessed using the advanced Data Processing Assistant for Resting-State fMRI (DPARSF^1^) (Chao-Gan and Yu-Feng, 2010; Yan et al., 2016), which is based on Statistical Parametric Mapping (SPM^2^). Specifically, the first 10 time points were discarded to suppress the equilibration effects and accommodate subjects’ adaptation to the environment. The remaining volumes were slice timing corrected, realigned, and spatially normalized to the MNI template and resampled to 3 × 3 × 3 mm^3^. Then, nuisance signals including cerebrospinal fluid, white matter, and head motion parameters and their derivatives based on the Friston 24-parameter model were regressed out in order to control the potential influence of physiological artifacts (Friston et al., 2015). In order to better remove head motion effects, each bad time point was regarded as a regressor. The bad time points were defined as volumes with framewise displacement (FD, Jenkinson) > 0.2 mm, as well as the two volumes succeeding and one volume preceding any such. The Jenkinson FD (Jenkinson et al., 2002) was used due to its consideration of voxel-wise differences in motion in its derivation (Yan et al., 2013). Additionally, linear and quadratic trends were also included as regressors, since the BOLD signal exhibits low-frequency drift. Finally, we conducted spatial smoothing with 4 mm full width at half maximum Gaussian kernel and bandpass temporal filtering (0.01–0.1 Hz).

### Dynamic Network Construction

Large-scale networks were established according to Power’s template, 264 regions are widely distributed across the whole brain and include fourteen large-scale networks (Power et al., 2011). Considering that signal intensity may be poorer in certain locations of the brain, 11 nodes in locations of lower BOLD signal were identified and discarded by using Wig’s approach (Wig et al., 2014); thus, 253 nodes were reserved. A sliding-window method was used to investigate the dynamic fluctuation of FC in each window. A time series of each ROI was acquired by averaging the BOLD signals from all voxels in this region, and the FC strength was examined by calculating the Pearson correlation of the time series between pairs of regions. Small and large windows were associated with a narrow range of network flexibility values across the brain, while medium windows (75 s, 100 s) uncovered a wide range of network flexibility (Telesford et al., 2016). Meanwhile, given that one TR is 2 s in this study and more windows are needed to track dynamic trajectories, window length was therefore set to 50 TRs, and the time gap of each window was set to 1TR, yielding 183 overlapping time windows to capture the temporal fluctuations of FC during a short period; next, Fisher’s z-transformation was applied to improve normality.

### Multilayer Community Detection

To explore the temporal evolution of modular structure in dynamic FC, we used a generalized Louvain algorithm to find the putative functional modules that based on the optimization of the modularity quality function (Telesford et al., 2016). Considering the fact that the multiple adjacency matrices correspond to the consecutive single time window, we connected the adjacent time windows by adding interlayer connections between each node and itself in the adjacent window (Mucha et al., 2010; Bassett et al., 2011). More details about this algorithm was provided in the Supplementary Material.

### Recruitment and Integration

Results of the multilayer community detection are shown in a partition matrix (nodes × layers), whose elements represent the community assignment of brain region *i* in layer *l*. The multilayer modularity quality function simultaneously and optimized assigns brain regions to communities in all layers so that community labels are consistent across layers, thus avoiding the community matching problem (Mucha et al., 2010; Bassett et al., 2011). According to the optimal community partition, module allegiance provides a summary of the consistency with which ROIs are assigned to communities (Bassett et al., 2015; Chai et al., 2016). To measure the dynamic role of each region within and between networks, we used the module allegiance matrix to compute two coefficients: the dynamic network integration and the dynamic network recruitment (Bassett et al., 2015; Mattar et al., 2015). Recruitment was defined as the probability of a region assigned to the same community with other regions from the same network, while integration was defined as the probability of a region being assigned to the same community as regions in other networks (Supplementary Material).

### Correlation Analyses and Independent *t*-Test

To identify how the brain network dynamics may be related to individual creative cognition, we calculated the Pearson correlation between recruitment and integration (including the region, network, and whole-brain levels) and the AUT score across all individuals, with gender, age, Raven’s score, and mean FD being regressed out as covariates. The results surviving FDR (*p* < 0.05) correction are reported. In addition, we calculated independent *t*-test to explore gender differences in creativity and intelligence scores.

## Results

### AUT Performance

Table 1 shows demographic information and Pearson correlation regarding all measures. There is no close correlation between Raven’s score and AUT performance (originality, *r* = 0.07, *p* = 0.18; flexibility, *r* = 0.06, *p* = 0.24; fluency, *r* = 0.08, *p* = 0.13; total score, *r* = 0.08, *p* = 0.15). We also found a significant gender difference: an independent *t*-test indicated that females showed a higher AUT score than males (originality, *t* = 2.68, *p* < 0.01; flexibility, *t* = 2.02, *p* < 0.05; fluency, *t* = 2.30, *p* < 0.05; total score, *t* = 2.49, *p* < 0.05), although there was no difference in Raven’s score (*t* = 1.16, *p* = 0.25). AUT and Raven’s score were both compliance with normal distribution.

**Table 1:** Demographic information and Pearson correlation.

Measures	Mean(*SD*)	Age	Raven	Originality	Flexibility	Fluency	AUT total score
Gender (166 males)							
Age	19.98(1.27)	–					
Raven	66.04(3.50)	–0.026	–				
Originality	10.30(4.64)	–0.080	0.070	–			
Flexibility	7.60(2.54)	–0.011	0.062	0.820^∗∗^	–		
Fluency	11.06(4.68)	–0.045	0.079	0.922^∗∗^	0.879^∗∗^	–	
AUT total score	28.96(11.42)	–0.053	0.075	0.968^∗∗^	0.917^∗∗^	0.981^∗∗^	–


### Correlation of Recruitment With AUT Performance

**FIGURE 1:**
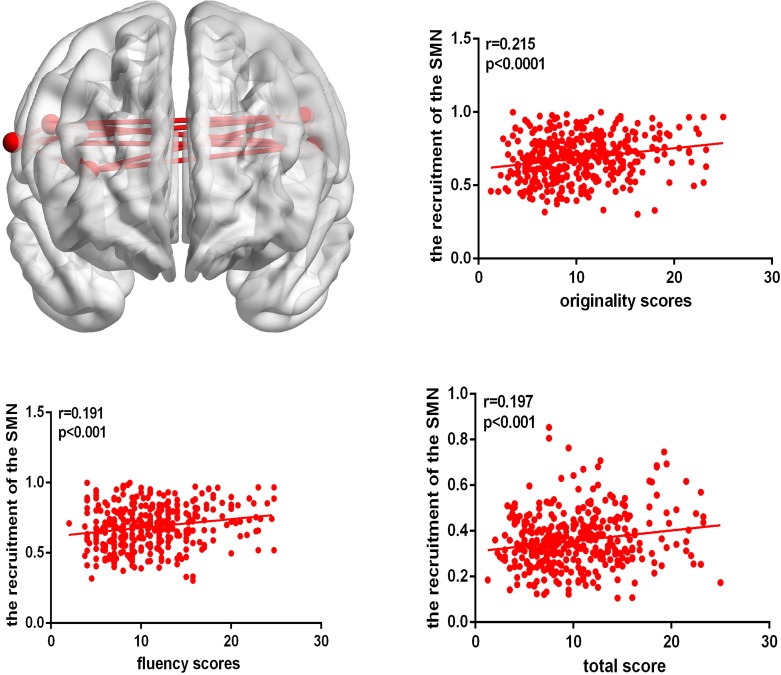
The brain map shows the recruitment of the SMN. The correlation map shows that the recruitment of the SMN is positively correlated with AUT originality, fluency, and total score.

After controlling for age, gender, Raven’s score, and mean FD, the recruitment of several nodes showed a positive close correlation with AUT performance among the 253 nodes, Interestingly, most of these nodes were located in the sensory/somatomotor network (SMN). Specifically, the recruitment of three nodes from the SMN and one node from the visual network showed positive correlation with originality score; the recruitment of three nodes from the SMN displayed positive correlation with both fluency and total scores. However, the recruitment of one node from the DMN showed a negative correlation with originality and fluency scores, as shown in Table 2. Meanwhile, at the network level, we also found that the recruitment of the SMN displayed significant correlations with originality, fluency, and total scores (originality, *r* = 0.215, *p* < 0.0001; fluency, *r* = 0.191, *p* < 0.001; total score, *r* = 0.197, *p* < 0.001; FDR corrected), as shown in Figure 1.

**Table 2:** The recruitment and integration of nodes correlated with AUT score (FDR corrected).

	Node index	Anatomical automatic labeling	*r*	*p*
Originality (recruitment)				
	44	Postcentral_R (SMN)	0.200	1.11 × 10^-4^
	45	Postcentral_L (SMN)	0.227	1.07 × 10^-5^
	46	Postcentral_R (SMN)	0.196	1.46 × 10^-4^
	117	Temporal_Mid_L (DMN)	–0.174	8.10 × 10^-4^
	160	Lingual_L (Visual network)	0.186	3.21 × 10^-4^
Fluency (recruitment)				
	42	Postcentral_L (SMN)	0.197	1.44 × 10^-4^
	44	Postcentral_R (SMN)	0.191	2.24 × 10^-4^
	45	Postcentral_L (SMN)	0.210	4.98 × 10^-5^
	117	Temporal_Mid_L (DMN)	–0.178	5.87 × 10^-4^
Total score (recruitment)				
	42	Postcentral_L (SMN)	0.201	9.77 × 10^-5^
	44	Postcentral_R (SMN)	0.195	1.63 × 10^-4^
	45	Postcentral_L (SMN)	0.216	2.91 × 10^-5^
Originality (integration)				
	79	Lingual_L (DMN)	0.202	9.53 × 10^-5^
	117	Temporal_Mid_L (DMN)	0.186	3.22 × 10^-4^


### Correlation of Integration With AUT Performance

**FIGURE 2:**
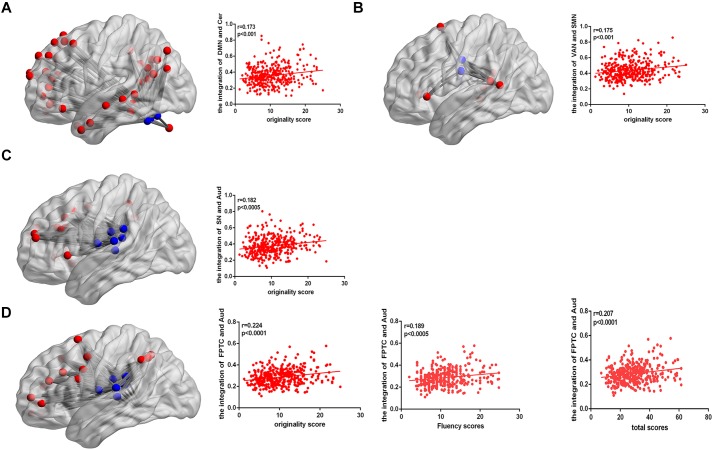
**(A)** The connectivity map shows the integration of the DMN and cerebellum. The correlation map shows that the integration of the DMN and cerebellum is positively correlated with AUT originality score. **(B)** The connectivity map shows the integration of the VAN and SMN. The correlation map shows that the integration of the VAN and SMN is positively correlated with AUT originality score. **(C)** The connectivity map shows the integration of the SN and Aud. The correlation map shows that the integration of the SN and Aud is positively correlated with AUT originality score. **(D)** The connectivity map shows the integration of the FPTC and Aud. The correlation map shows that the integration of the FPTC and Aud is positively correlated with AUT originality, fluency and total scores.

After controlling gender, age, Raven’s score, and mean FD, the integration of two nodes (node number defined in the Power template: 79 and 117) from the DMN showed a positive close correlation with originality scores (node 79, *r* = 0.202, *p* < 0.0001; node 117, *r* = 0.186, *p* < 0.0005; FDR corrected), as shown in Table 2. We also calculated the integration between pairs of networks, originality score showed a positive correlation with the integration of the DMN and cerebellum (*r* = 0.173, *p* < 0.001), the ventral attention network (VAN) and SMN (*r* = 0.175, *p* < 0.001), the salience network (SN) and auditory network (Aud) (*r* = 0.182, *p* < 0.0005), and the frontoparietal task control (FPTC) network and Aud (*r* = 0.224, *p* < 0.0001), corrected by FDR. Fluency and total scores both displayed positive correlations with the integration of the FPTC and Aud (fluency, *r* = 0.189, *p* < 0.0005; total scores, *r* = 0.207, *p* < 0.0001; FDR corrected); flexibility score was also related to the integration of the FPTC and Aud (*r* = 0.174, *p* < 0.001, uncorrected), as shown in Table 3 and Figure 2.

**Table 3:** The integration between pairs of networks correlated with AUT score (FDR corrected).

AUT score	Pairs of network	*r*	*p*
Originality			
	DMN-Cer	0.173	8.56 × 10^-4^
	VAN-SMN	0.175	9.75 × 10^-4^
	SN-Aud	0.182	4.43 × 10^-4^
	FPTC-Aud	0.224	1.02 × 10^-5^
Fluency			
	FPTC-Aud	0.189	2.50 × 10^-4^
Total score			
	FPTC-Aud	0.207	5.85 × 10^-5^


## Discussion

As far as we know, this is the first investigation exploring whether the dynamic reorganization of networks is correlated with verbal creativity using dynamic multilayer community detection approaches. We associated the dynamic of resting-state functional brain networks at three different levels (the regional level, the network level, and the whole-brain level) with the AUT performance to reveal the brain mechanisms of creativity. We found that the recruitment of bilateral postcentral gyrus in SMN showed a positive close correlation with AUT performance. Meanwhile, at the network level, we also found that the recruitment of the SMN displayed a significant correlation with originality, fluency, and total scores. For integration, the left lingual gyrus and left MTG in the DMN showed a positive close correlation with originality scores. Furthermore, we revealed that 4 between-network integrations were clearly related to the AUT performance. Specifically, the integration of DMN and cerebellum, FPTC and Aud were involved. These results suggested that the dynamic communication among the brain networks concerning spontaneous thought, cognitive control, and external information inputs was significant for generating creative ideas. Meanwhile, previous research indicated the dynamic communication among the networks has been association with cognitive flexibility (Dietrich, 2004), and our results further demonstrated the relationship between verbal creativity and cognition flexibility.

**FIGURE 3:**
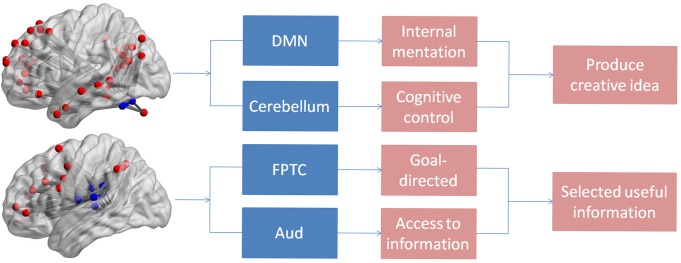
The figure shows the individual and integration functions of the brain networks.

In the present study, we found individuals with greater creativity performance displayed stronger integration in DMN network including the left lingual and left MTG. The result might suggest individuals should retrieval previous memory during the process of creativity tasks. The medial temporal lobe memory network may facilitate generation of creative ideas by establishing association between old and new information (Ellamil et al., 2012). Previous studies showed that the lingual gyrus was favorably activated in novel processes (Jung et al., 2010). At the network level, we found that verbal creativity performance was positively correlated to the integration of DMN and cerebellum. It is well known that DMN activity was mainly linked to internal mentation, such as mind wandering (Ionta et al., 2014) and future imagination (Stawarczyk and D’Argembeau, 2015). Thus activation of the DMN was involved in the process of generating creative ideas. Existing studies exposed that the cerebellum was relevant to cognitive control including visual attention and working memory (Brissenden et al., 2015). Meanwhile, Pidgeon et al. (2016) reported the cerebellum was also linked with the production of creativity improvisation and greater creative imagining in pictures (Pidgeon et al., 2016). The result of the integration of two networks being closely related to creativity showed the cooperation between spontaneous thought and cognitive control. It was contributed to generating more creative ideas that were consistent with current problems, as shown in Figure 3. Besides, increased functional connectivity between default network hubs and regions involved cognitive control was related to openness (Adelstein et al., 2011), and openness can strongly predicts performance on creative thinking tasks (Silvia et al., 2008), which could indirectly demonstrate the stronger correlation of DMN and cerebellum linked up with greater performance in verbal creative tasks. In addition, we also found that integration of FPTC and Aud were positively correlated with verbal creative performance, showing that the dynamic connectivity between the FPTC and Aud was associated with verbal creativity. The activity of the FPTC was related to many high-control cognitive functions which required an externally goal-directed process (Anselm et al., 2013). The integration of FPTC and Aud indicated that the stronger association of the goal-directed and auditory networks might facilitated selection of surrounding information from ears, as shown in Figure 3.

Regarding recruitment, we found that the recruitment of several nodes showed a positive close correlation with AUT performance among the 253 nodes; interestingly, most of these nodes were located in the SMN. Specifically, the recruitment of 3 nodes from the SMN, including the bilateral postcentral gyrus, showed a positive correlation with originality, fluency and total scores. These regions might benefit to emulating usage methods of items in somatosensory areas, which could be profitable for generating novel answers in AUT (Cousijn et al., 2014). In addition, at the network level, we found that the recruitment of the SMN displayed significant correlation with originality, fluency, and total scores. It could be concluded that high creative performance might require the SMN to remain stable at resting states, communicating mainly with other regions within the same network, lack of association with other network (Shi et al., 2018a). The results also explained why FC of the SMN and other brain networks has rarely been found in previous studies.

Prior research about verbal creativity have been interested in the gross network characteristics (Beaty et al., 2014) or static functional connections (Gao et al., 2017), thereby ignoring the dynamic reorganization of the brain networks. Here, we used dynamic neuroscience methods to track changes in the recruitment and integration of networks during resting states, and identified the correlation between the dynamic reorganization of brain networks and verbal creativity. Furthermore, we found that creativity was related to multiple cognitive processes, including memory retrieval, imaginative process, and cognitive control, which suggested that it is possible to improve creative performance by training these basic cognitive processes. For example, if memory was improved, you would be able to more quickly extract previous memory and establish links between old memory and new information. In the same vein, if the ability of cognitive control was trained, you would accurately suppress unrelated information and mind. These process are important for generating novel ideas (Arden et al., 2010). Meanwhile, prior studies revealed significantly reduced FC between the cerebellum and the DMN in depression (Liu et al., 2012). On the contrary, we found verbal creativity performance was positively correlated to the integration of DMN and cerebellum, which provides the possibility of increasing functional connectivity between the cerebellum and the DMN through creativity trains to treat depressed patients.

This study also had some possible limitations. First, we used dynamic community detection algorithm to integrate brain regions into a coherent activation community. However, due to the inherent challenges of heuristic algorithms and fMRI data, node allocation at the individual subject level is still a statistical process with certain degree of uncertainty (Braun et al., 2015). Second, task-based fMRI is more meaningful for studying the dynamic variation of brain mechanism than resting-state fMRI. In the future, it would be necessary to research the dynamic variation of networks during creativity tasks. Finally, we used a single test (AUT) to represent a multidimensional conception (creativity), which was also insufficient. Future researches would employ multidimensional approaches of measuring creativity to find the comprehensive brain mechanism during creativity tasks.

## Conclusion

In summary, this is the first investigation to study the relationship between the dynamic reorganization of brain networks and verbal creativity using dynamic community detection. Our study found that the integration of left lingual and left MTG in DMN regions and the integration of DMN and cerebellum, FPTC and Aud showed positive correlation with verbal creativity performance. In addition, the recruitment of bilateral postcentral gyrus from the SMN and the recruitment of the SMN showed positive correlation with verbal creative performance. These findings provided direct evidence that verbal creativity was related to the dynamic variation of neural mechanism during resting-state, extending past research on the neural mechanism of verbal creativity. At the same time, this result brought about new perspectives for creative training and rehabilitation training of depression.

## Ethics Statement

This study was approved by the Institutional Review Board of Southwest University Imaging Center for Brain Research, all participants signed the written informed consents and received payment for their participation.

## Author Contributions

JQ and WY were responsible for design the experiments. QF was responsible for writing the article. LH and XW was responsible for analyzing the data. YZ was responsible for collecting the data.

## Conflict of Interest Statement

The authors declare that the research was conducted in the absence of any commercial or financial relationships that could be construed as a potential conflict of interest.

## Footnotes

1 http://www.rfmri.org/DPARSF

2 http://www.fil.ion.ucl.ac.uk/spm
