# Supplementary material for: Verbal Creativity Is Correlated With the Dynamic Reconfiguration of Brain Networks in the Resting State
Source: Front Psychol. 2019 Apr 24;10:894. doi: 10.3389/fpsyg.2019.00894 (PMC6491857; doi:10.3389/fpsyg.2019.00894)
Supplement: Supplementary file 1 [file Table_1.DOC]

**Supplementary Material**

**Multilayer Community Detection**

For each participant, we used a generalized Louvain algorithm to find the putative functional modules that based on the optimization of the modularity quality function . Specifically, we used a generalized Louvain-like method originally developed to optimize a single-layer modularity quality function , and then extended to optimize the following multilayer modularity quality function :


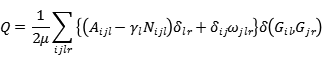


In this function, *Q* is the multilayer modularity index,
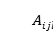
 is the connection strength of region *i* and *j* in layer *l*,
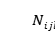
 is the corresponding elements in a null model matrix,
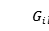
 represents the community assignment of node *i* in layer *l*,
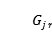
 represents the community assignment of node *j* in layer *r*,
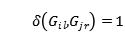
 if
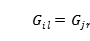
 and it equals 0 otherwise,
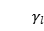
 is the structural resolution parameter of layer *l*, the temporal resolution parameter
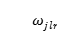
 is the connection strength between node *j* in layer *l* and node *j* in layer *r*, *μ* is the total edge weight in the network,
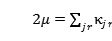
, the strength of node *j* in layer *l* is
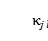
=
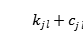
, the intra-layer strength of node *j* in layer *l* is
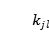
, and the inter-layer strength of node *j* in layer *l* is
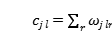
. We used the Newman-Girvan null model in each layer:
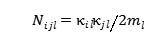
, where
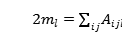
 is the total edge weight in layer *l*. The structural resolution parameter determines the number of modules within a given layer, and the temporal resolution parameter controls the consistency of modules detected across layers . In order to avoid the deviation from the results of multilayer community detection , while considering the simplification of calculations and the consistency with previous research, we used the common default values that set
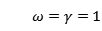
 . In addition, due to the community detection algorithm is vulnerable to near-degeneracies and stochastic elements in this algorithm, the output usually varies from run to run. We therefore performed the multilayer community detection algorithm 100 times to yield 100 optimized partitions of the multilayer modularity .

**Module Allegiance**

Module allegiance provides a summary of the consistency with which ROIs are assigned to communities . For each participant during each optimization, each element
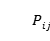
 of their module allegiance matrix *P* gives the relative frequency that nodes *i* and *j* were assigned to the same community across time windows. The element
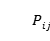
 will be 1 if nodes *i* and *j* are always in the same community and 0 if they are never in the same community. Values of
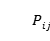
 are then averaged across optimizations for each participant.

**Recruitment and Integration**

We use the module allegiance matrix to assess the dynamic roles of brain systems. To quantify the dynamic role of a region within one of these systems, we use the module allegiance matrix to compute two coefficients: the dynamic network recruitment and the dynamic network integration . The recruitment coefficient of region *i* with respect to system *S*, is defined as:


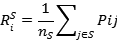


Where
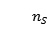
 is the size of system *S*, calculated as the number of regions in *S*. Accordingly,
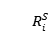
 corresponds to the average probability that the
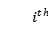
 region is in the same community as other regions of the system *S*. A region with high recruitment to system *S* is one that tends to be found in system *S* across time windows. The integration coefficient of region *i* with respect to system *S*, is defined as:


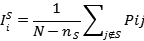


where *N* is the total number of brain regions. Accordingly,
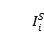
 corresponds to the average probability that the
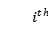
 region is in the same community as regions from systems other than *S*. A region in system *S* with high integration is one that tends to be found in systems other than its own across time windows. Both coefficients were characterized at the subsystem and global levels, after averaging values across optimizations for each participant.
